# Awake Single-Stage Bilateral Clavicle Surgeries Under Bilateral Clavipectoral Fascial Plane Blocks: A Case Report and Review of Literature

**DOI:** 10.7759/cureus.20537

**Published:** 2021-12-20

**Authors:** Kartik Sonawane, Saisrivas Dharmapuri, Shlok Saxena, Tuhin Mistry, J. Balavenkatasubramanian

**Affiliations:** 1 Anesthesiology, Ganga Medical Centre and Hospitals Private Limited, Coimbatore, IND

**Keywords:** clavicle surgery, modified clavipectoral fascial plane block, awake clavicle surgery, clavipectoral fascial plane block, fascial plane block, bilateral clavicle fracture

## Abstract

The clavicle is a frequently fractured bone with an infrequent bilateral occurrence. Regional anesthesia (RA) for clavicle surgeries is always challenging due to its complex innervation arising from the two plexuses (cervical and brachial). Various RA techniques described for clavicle surgeries include plexus blocks, fascial plane blocks, and truncal blocks. Plexus blocks are associated with undesirable effects, such as phrenic nerve blockade and paralysis of the entire upper limb, limiting their application for bilateral regional clavicle surgeries. The clavipectoral fascial plane block (CPB) is a novel, procedure-specific, phrenic-sparing, and motor-sparing RA technique that can provide anesthesia or analgesia for clavicle surgeries. The decision to use the CPB and/or other RA techniques may depend on the site of clavicle injury or variations in clavicular innervation.

We report a case of single-stage bilateral clavicle surgery successfully managed with a bilateral CPB alone using ultrasound guidance and landmark guidance separately. The patient was kept awake and comfortable throughout the surgery.

In conclusion, CPB can be an effective alternate RA technique in avoiding undesired side effects of more proximal techniques such as phrenic nerve involvement and motor blockade of upper limbs. Landmark-guided CPB can be an alternative with equianalgesic efficacy as of ultrasound-guided CPB in resource-poor or emergency settings.

## Introduction

Clavicle fractures (CFs) are frequently encountered in emergency and operating room settings, accounting for 2.6% of all fractures [1]. The most common site for CF is in its middle third, with an incidence of 5-10% in young patients [2-4]. However, bilateral CFs are extremely rare [5-8], with an incidence of only 0.011-0.017% [9], comprising <0.5% of all CF.

Most CFs heal with good functional results after early surgical intervention. Therefore, the surgical fixation of bilateral CF in a single-stage depends entirely on the surgeon’s approach, taking into account the type/site of fractures, associated injuries, and expected functional outcomes of the patient. Such surgery would require general anesthesia (GA) with or without regional anesthesia (RA) due to limitations in administering bilateral plexus blocks.

Regional anesthesia for clavicle surgeries is quite challenging because of the complex innervations contributed by cervical and brachial plexuses [10]. RA options for the clavicle comprise plexus blocks, truncal blocks, or fascial plane blocks [10]. Plexus blocks involve the cervical plexus (superficial cervical plexus block or selective supraclavicular nerve block) with or without brachial plexus (interscalene or selective superior trunk) blocks. While the plexus blocks are associated with motor weakness of the upper extremity, the efficacy of the truncal blocks like erector spinae plane block [11] or the pectoral nerve blocks [12-13] (PEC1 and PEC2) is yet to be determined. The clavipectoral fascial plane block (CPB) can provide anesthesia or analgesia for CF, eliminating the disadvantages of plexus blocks [14]. Although CPB is administered under ultrasound guidance, it can be given using landmark guidance with equianalgesic efficacy. Since this case report, a handful of additional case reports demonstrated and supported the effectiveness of CPB for clavicle surgery [15-18].

This case report is the first of its kind to document a single-stage awake bilateral clavicle surgery performed under bilateral CPB alone, using ultrasound and landmark guidance separately, with equivocal results. We also discussed modifications required when performing CPB in a comminuted type of CF and limitations of CPB in certain scenarios due to insufficient drug spread leading to inadequate analgesic coverage. The patient provided written informed consent for undergoing the procedure, sharing case-related images (with hidden identity), and publishing this case report.

## Case presentation

**Figure 1:**
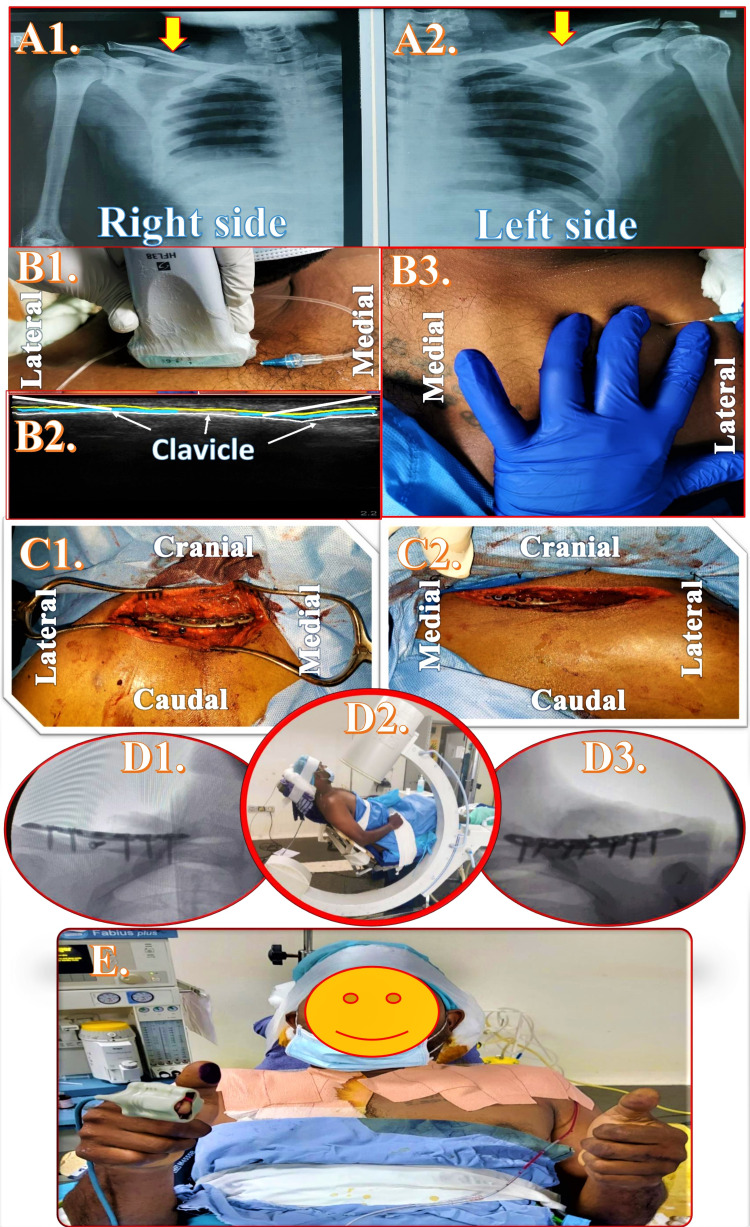
Radiographic and clinical images of the bilateral clavipectoral fascial plane block and bilateral clavicle fracture surgery A1, A2: Radiographic images of right and left clavicle fracture; B1, B2: Performance of ultrasound-guided clavipectoral fascial plane block; B3: Performance of landmark-guided clavipectoral fascial plane block; C1, C2: Surgical fields of bilateral clavicle surgery with implants in situ; D1, D2, D3: Patient positioning during surgery and intraoperative radiographic pictures; E: Smiling patient able to lift both upper extremities immediately after surgery

A 31-year-old, healthy male driver was admitted to the emergency room with a bilateral Allman type I clavicle fracture (Figure 1, panels A1-1A2) due to direct trauma from a bicycle handlebar following a self-skid. He had no associated brachial plexus or chest injury. He was scheduled for a single-stage open reduction and internal fixation of both clavicles with screws and locking plates under bilateral CPB.

Preoperatively, the patient was premedicated with intravenous pantoprazole 40 mg, ramosetron 0.3 mg, and midazolam 2 mg. A local anesthetic (LA) mixture was prepared using 20 ml of 2% lignocaine with epinephrine, 20 ml of 0.5% bupivacaine, 20 ml of normal saline, and 8 mg of dexamethasone. The CPB was performed using ultrasound on the right side and landmark guidance (Figure 1, Table 1) on the left side.

**Table 1:** Descriptive comparison between ultrasound-guided and landmark-guided clavipectoral fascial plane block CPB: Clavipectoral fascial plane block, LA: Local anesthetic

	Ultrasound-guided CPB (US-CPB)	Landmark-guided CPB (LM-CPB)
Local anesthetic (LA):	20 ml of 2% lignocaine with epinephrine + 20 ml 0.5% of bupivacaine + 20 ml of normal saline + 8 mg dexamethasone	
LA volume:	10 ml for medial and lateral injections 10 ml around the fracture site (in modified approach) 10 ml for the skin infiltration around the incision site (if required)	
Patient position:	Supine with head turned to the opposite side	
Probe/Landmarks:	High-frequency linear probe kept, Sagittally over the medial and lateral ends of the clavicle, OR Transversely along the length of the clavicle	Palpating medial and lateral ends of the clavicle using fingers
Needle:	1.5 inch 23G hypodermic needle	
Needle direction and LA deposition:	With the probe in the sagittal plane: The needle is inserted in-plane from caudal-to-cranial direction depositing LA between clavipectoral fascia and periosteal collar. OR With the probe kept transversely along the clavicle: First moving probe medially towards the medial end, the needle is inserted in-plane from medial-to-lateral direction depositing LA above periosteal collar from medial end to the midpoint of the clavicle. Then, moving probe laterally towards the lateral end, the needle is inserted in-plane from lateral-to-medial direction depositing LA from lateral to the midpoint of clavicle above the periosteal collar. Third injection is required in a modified approach where the probe is kept over the fracture site, and LA is deposited around it under vision.	First injection: Medial end of the clavicle is palpated using a finger. LA is deposited over the medial end from the medial-to-lateral direction after hitting the bone. Second injection: Lateral end of the clavicle is palpated using a finger. LA is deposited over the lateral end from the lateral-to-medial direction after hitting the bone. Third injection: LA is deposited around the fracture site after hitting the bone on either side of the fracture site
Analgesic coverage:	Osteotomal innervations of the whole clavicle due to LA distribution over periosteum and dermatomal innervation may get involved due to the diffusion of the LA.	
Rescue technique:	Separate skin infiltration is required if supraclavicular nerves are not covered	
Advantages:	Completely motor-sparing technique; simple to learn, administer, or teach Less painful due to hypodermic needle	Completely motor-sparing technique; simple to learn, administer, or teach; economical as no requirement of special equipment like ultrasound or special skills; suitable for remote, poor resource, or emergency setting; less painful due to hypodermic needle
Disadvantage:	Limited extent of field block; may be ineffective in revision surgery, implant removal surgery, comminuted fractures, or nonunion/malunion surgery; not economical due to requirement of equipment like ultrasound and special skills in regional anesthesia	Limited extent of field block; may be ineffective in revision surgery, implant removal surgery, comminuted fractures, or nonunion/malunion surgery

Ultrasound-guided right CPB

With the patient in the supine position and head turned to the left side, a high-frequency linear ultrasound probe (Sonosite HFL38x/13-6 MHz; Fujifilm SonoSite, Bothell, WA) was positioned transversely over the right clavicle (Figure 1, panel B1). After identifying hyperechoic clavicular shadow under ultrasound (Figure 1, panel B2), a 1.5-inch hypodermic (23G) needle was inserted in-plane from the medial and lateral ends of the probe to deposit LA solution over the periosteal collar.

Landmark-guided left CPB

With the patient in the same position and head turned to the right side, the fingers were kept over the medial and lateral ends of the clavicle (Figure 1, panel B3). A 1.5-inch hypodermic (23G) needle was inserted adjacent to the palpating fingers to deposit LA solution after making contact with the periosteum of the left clavicle.

We used three injections for each technique (ultrasound-guided and landmark-guided) with a volume of 10 ml/injection: two injections over the medial and lateral ends of the clavicle and a third injection over the fracture site.

Block outcome

Before the CPB, the patient had restricted shoulder joint movements on both sides due to severe pain with a score of 7/10 on a numeric rating scale (NRS). Every five minutes after CPB, the sensory blockade was assessed using pinprick and cold sensations on a three-point scale (2 = normal sensation, 1 = decreased sensation, and 0 = no sensation) in the surgical dermatomal area (C3-C7). Twenty minutes after CPB, his pain scores decreased to 0/10 on NRS. Thirty minutes after CPB, the patient was transferred to the operating room after confirming the complete loss of pinprick and cold sensations over the proposed surgical sites.

Intraoperatively, the patient was positioned in a beach chair position (Figure 1, panel D2). The surgeon rechecked the sensory block using toothed forceps before the incision. The patient’s comfort was ensured by communicating intermittently with him and addressing his concerns or complaints. While undergoing surgery on the right clavicle, the patient complained of mild pain over the right shoulder region due to an unexpected lateral extension of the incision (beyond the dermatomal coverage area) by the surgeon. An intravenous bolus of 60 micrograms of fentanyl was administered, followed by an additional LA infiltration in the spared cutaneous area by the surgeon. Intravenous paracetamol 1 g, ketorolac 30 mg, and dexamethasone 8 mg were given as part of multimodal analgesia. The rest of the intraoperative course was uneventful without any need for additional analgesics, deep sedation, or conversion to GA. The patient remained comfortable, pain-free, and awake throughout the procedure of about 3.5 hours with blood loss of around 300 ml. Immediately after surgery, the patient could lift both of his upper extremities without any pain (0/10 on NRS) with a smile on his face (Figure 1, panel E).

Postoperatively, the patient was monitored in the recovery room for two hours and advised to resume oral intake within an hour of surgery. A multimodal analgesia protocol was provided for postoperative pain that included oral acetaminophen 1 g four times daily, aceclofenac 100 mg twice daily, and pregabalin 75 mg at bedtime. He was discharged on the second postoperative day with a pain score of 2/10 on NRS without any requirement of rescue analgesia or opioids.

Review of literature

The application of the RA to clavicle surgery is moving towards a more procedure-specific approach with greater clarity of its complex innervation. A recent cadaveric study by Leurcharusmee et al. described the contribution of the subclavian, lateral pectoral, and supraclavicular nerves to the innervation of the clavicle [19]. The current literature reinforces the use of RA for CF to reduce perioperative opioid consumption and promote enhanced recovery and discharge. Dooley et al. recommended using RA due to its superior pain relief and safeguarding from GA-associated complications [20]. Likewise, Ryan et al. described RA as effective, safe, and time conserving [21]. Proximal brachial plexus block has been a standard practice for anesthesia or analgesia for CF. Neha Gupta et al. successfully performed clavicle surgeries exclusively under RA with interscalene block (ISB) combined with superficial cervical plexus block (SCPB) [22]. A more precise approach of the selective upper trunk block, described by Gurumoorthi et al. for CF fractures demonstrated similar outcomes [23]. Olofsson et al. found a substantial decrease in total opioid consumption with ISB as an analgesic block in patients receiving GA for clavicle surgery [24].

Valdes et al. revolutionized surgeries for CF with the introduction of CPB in 2017 [25]. This fascial plane block targets the sensory nerves to the clavicle that traverse the clavipectoral fascia. Numerous case reports have emerged since its description (Table 2). Ince et al. successfully anesthetized a patient using CPB with skin infiltration and suggested the safe and effective use of CPB to provide anesthesia for clavicle surgeries as an alternative to ISB [15]. Atalay et al. added evidence to support the use of CPB for medial end clavicle fracture to provide anesthesia and analgesia as an alternative to the ISB [18,26]. Kukreja et al. further highlighted the motor-sparing and phrenic-sparing effects as added benefits of this more distal block [14]. Similar observations were appreciated by Ueshima et al. for lateral end clavicle fracture [16]. Magalhães et al. noticed good analgesia with CPB when used as an adjunct to GA in clavicle surgery [27]. Furthermore, the utility of CPB in breast surgery was explained by Tulgar et al. [28] and in the cardiovascular implantable electronic device placement by Metinyurt et al. [29]. Effective analgesia can be achieved with CPB when combined with SCPB as per Rosales et al. [30] or and supraclavicular nerve block (SCNB) as per Gonçalves et al. [31].

**Table 2:** Literature analysis of clavipectoral fascial plane block between the years 2019-2021 CPB: Clavipectoral fascial plane block, GA: General anesthesia, RA: Regional anesthesia, SCPB: Superficial cervical plexus block, ISB: Interscalene block, SCNB: Supraclavicular nerve block

Study	Year	No. of cases	Age/Sex of patient	Fracture site/ surgery	Anesthesia	LA type and Volume	Conclusion
Ince I. et al. [15]	2019	01	51/M	Left midshaft clavicle fracture	CPB + Local infiltration over the incision site	30 ml of LA mixture (1:1 0.5% bupivacaine+2% lidocaine)	CPB can be an alternative to interscalene block
Ueshima H. et al. [16]	2019	01	57/M	Emergent percutaneous coronary intervention + fixation of right distal end clavicle fracture	GA + CPB	20 ml of 0.25% levobupivacaine	CPB can provide effective analgesia and also can be alternate to brachial plexus block
Yoshimura M. et al. [17]	2019	02	37/M and 71/F	Fixation of the clavicle fracture	GA + RA (CPB +SCPB)	15 ml of 0.375% levobupivacaine	CPB can be simple and safe for analgesia/anesthesia in clavicle fractures
Atalay Y. et al. [18]	2019	01	47/F	Fixation of the clavicle fracture	GA + RA (CPB +SCPB)	20 ml of 0.25% bupivacaine	CPB can be suitable for anesthesia, postoperative pain management, emergency pain management, biopsies, or curettage of the clavicle bone tumors
Atalay Y. et al. [26]	2020	05	18-37/M	Fixation of the clavicle fracture	GA + RA (CPB +SCPB)	20 mL of 0.25% bupivacaine	CPB can be an alternative to interscalene block with analgesic effectiveness in clavicle fracture
Kukreja P. et al. [14]	2020	03	32/F, 14/M, and 19/F	1. Right distal clavicle excision 2. Implant removal right sternoclavicular joint 3. Left midshaft clavicle fracture fixation	1. CPB + continuous right ISB 2. GA + CPB 3. GA + CPB	1. 15 ml 0.5% ropivacaine for CPB 10 cc 0.5% ropivacaine bolus and 0.2% ropivacaine solution at 8 ml/hr 2. 15 ml 0.5% ropivacaine with 2 mg of dexamethasone 3. 10 ml 0.5% ropivacaine	CPB can be an effective alternative in avoiding motor-blockade and phrenic nerve paralysis due to proximal techniques and it does not carry the risk of pneumothorax
Magalhães J. et al. [27]	2020	04	?	Midshaft clavicle fracture fixation	GA + CPB	10 to 15 ml of levobupivacaine 0.4%	For combined anesthesia, a single puncture approach of CPB constitutes an excellent analgesic option
Tulgar S. et al. [28]	2020	01	72/F	Breast-conserving surgery for right breast tumor	GA + Modified CPB	A total of 40 mL LA (20 mL bupivacaine 0.5%, 10 mL lidocaine 2%, and 10 mL normal saline)	Adequate sensorial block was achieved at the outer quadrant of the breast with modified CPB
Metinyurt H. et al. [29]	2021	02	64/F and 37/M	Cardiac Implantable electronic device (CIED) implantation	CPB	15 ml of 0.5% bupivacaine for medial and lateral injections	CPB can be considered an alternative to other anesthesia techniques for CIED implantation
Rosales A. et al. [30]	2021	07	?	Midshaft clavicle fracture fixation	2 patients with CPB only and 5 patients with CPB + SCPB	?	CPB with or without SCPB provided effective and safe anesthesia and analgesia in clavicle surgery under GA or intravenous sedation
Gonçalves D. et al. [31]	2021	01	25/M	Implant removal right clavicle	CPB + SCNB	20 + 5 mL (1:1 - 0.75% ropivacaine+ 2% mepivacaine)	A combination of CPB + SCNB is safe and easy to perform and reduces risks of phrenic nerve block and upper limb paralysis

The current evidence for CPB is favorable for its use in clavicle surgeries to provide analgesia and anesthesia with additional advantages over the conventional plexus block. However, in the absence of randomized controlled trials, more research is needed to recommend CPB over brachial and cervical plexus blocks.

## Discussion

The worldwide incidence of CF varies between 24-71 per 100,000 population per year, which shows an increasing trend [4]. These injuries are common in young adults and children, mostly men <25 years of age [4]. Due to the associated high-energy trauma, CF usually occurs with other concomitant injuries. Bilateral CF mainly results from a compressive force across both shoulder girdles, direct blows to both shoulder girdles, or an indirect blow such as a fall onto the shoulder [5,8]. Due to the rare occurrence of bilateral CF, the possibility of a single-stage bilateral surgical intervention becomes even rarer. The surgeon’s preference for operating bilateral clavicles in a single-stage is mainly focused on avoiding the morbidity associated with prolonged conservative management and promoting early ambulation as such patients could not even perform activities of daily life like maintaining personal hygiene, eating or drinking, getting up from the bed without assistants, or lying on lateral or prone position. Evidence in the literature suggests a high risk for nonunion or shoulder dysfunction after nonoperative treatment in bilateral CF [32-33]. Eventually, the management of CF further requires an assessment of the patient's expectations and activity levels [34]. This report discusses the safe and effective use of bilateral CPB with two different modalities (ultrasound and landmark) for bilateral CF surgery while keeping the patient awake.

**Figure 2:**
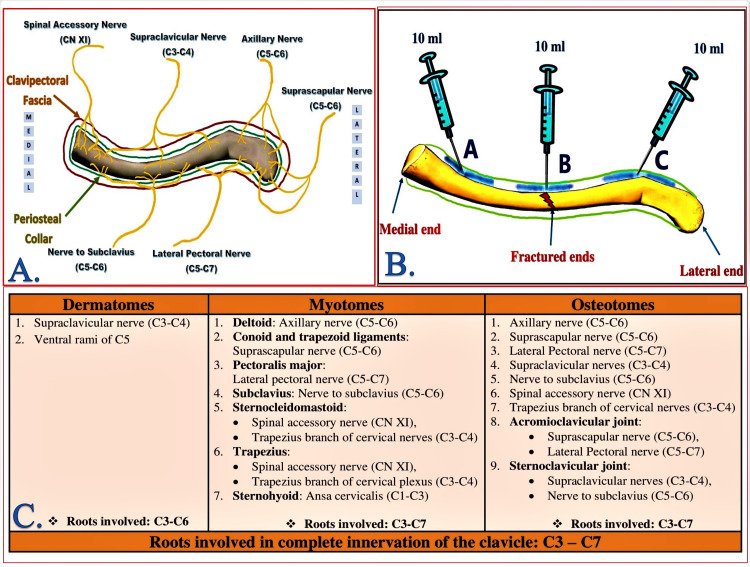
Innervation of the clavicle and injection pattern of landmark-guided clavipectoral fascial plane block A: Osteotomal innervation of the clavicle; B: Injection pattern of landmark-guided clavipectoral fascial plane block; C: Complete innervation of the clavicle (Source of A and C: Sonawane K, Dixit H, Balavenkatasubramanian J, Gurumoorthi P (2021) Uncovering secrets of the beauty bone: A comprehensive review of anatomy and regional anesthesia techniques of clavicle surgeries. Open J Orthop Rheumatol 6(1): 019-029. DOI: https://dx.doi.org/10.17352/ojor.000034)

Successful administration of RA in clavicle surgeries requires a thorough understanding of its complex innervations. The clavicle derives its sensory innervation from the cervical and brachial plexuses via various nerves in the vicinity that supply either the skin, muscles, joints, or ligaments [10]. The pain-generating structures in any clavicle surgery include the overlying skin and the richly innervated periosteum. The subcutaneous location of the clavicle negates the need for muscle relaxation intraoperatively. Therefore, the target innervations for RA in clavicle surgery should encompass dermatomes and osteotomes, with the least focus on myotomes (Figure 2, panels A, C).

Complex innervations contributed by two different plexuses make any individual block insufficient to achieve the desired results. Although the high-volume ISB often blocks the cervical plexus [35], combinations of RA techniques are recommended to avoid complications. Such combinations include ultrasound-guided ISB + SCPB [36], superior trunk block + SCPB [37], or SCUT (selective supraclavicular + upper trunk) block [38]. Sivashanmugam et al. considered the SCUT block as an effective site-specific RA strategy for clavicle surgery in their study on 70 patients [38]. Plexus blocks like ISB or SCPB can cause hemidiaphragmatic paresis due to associated blockade of the phrenic nerve that can cause detrimental effects in some patients with obstructive sleep apnea, obesity, or significant underlying lung disease [14]. ISB can also cause motor blockade of the upper extremity and other complications like Horner’s syndrome, local anesthetic systemic toxicity, total spinal anesthesia, nerve injuries [39-42], and adverse events like epidural or vertebral artery injections [43]. The associated risk of phrenic nerve involvement, sympathetic blockade, and upper extremity paralysis prohibits the application of plexus blocks bilaterally.

Clavipectoral fascial plane block can be administered bilaterally without the risk of any neural damage, phrenic nerve blockade, or upper limb paralysis to provide effective anesthesia or analgesia in clavicle surgeries [14,17]. The advantages of CPB include the ease of administration, an advanced safety profile in patients with respiratory diseases, and improved safety advantage due to superficial injection limited by the natural backstop (clavicle) that avoids injury to deeper structures [14]. It acts by blocking all the nerves running in the plane between the clavipectoral fascia and the periosteal collar of the clavicle. CPB can be safe and effective in trauma patients with rib fractures and pneumothorax, in whom GA can cause adverse effects [44]. The choice of the RA techniques for clavicle fracture (Table 3) depends on the many factors: fracture location (medial/lateral end), associated injuries, unilateral/bilateral fractures, nature of fracture (comminuted/noncomminuted/displaced/nondisplaced), type of surgery (recent trauma/old trauma/revision surgery), and respiratory parameters of the patient.

**Table 3:** Choices of available RA techniques as per the type of the clavicle surgeries A: Interscalene block + Superficial cervical plexus/supraclavicular nerve block, B: SCUT block (Selective supraclavicular nerve block + selective upper trunk block), C: Clavipectoral fascial plane block LA: Local anesthetics

Clavicle surgery	First choice RA	Second choice RA	Third choice RA	Comments
Unilateral clavicle fracture without scapula fracture	C	B	A	For medial end fracture surgery: An additional LA infiltration over the medial end periosteum and subcutaneous tissues is required.
Unilateral clavicle fracture with scapula fracture	A	B	C	
Unilateral comminuted fracture/ Revision surgery/ Implant removal surgery/ Non-union, mal-union surgery	B	A	C	
Bilateral clavicle fractures	C (bilaterally)	B (on one side) C (on another side)	B >A (on both sides) with low LA volume (5-7 ml). Staged block: one side block for first clavicle surgery followed by second side block for second clavicle surgery.	

Ultrasound-guided CPB involves two injections at either end of the clavicle and the deposition of LA in the plane between the clavipectoral fascia and the periosteal collar of the clavicle. Its analgesic coverage depends on adequate LA distribution around the clavicle to block all nerves that pierce the clavipectoral fascia before supplying the clavicle. Anatomical factors that can affect LA spread include the integrity of the clavipectoral fascia and the potentiality of the space between it and the periosteal collar of the clavicle [10]. The possible violation of the fascial integrity in conditions like comminuted or displaced CF can impair the LA propagation, resulting in a sparing effect [10]. We propose a modification in CPB that includes an additional injection over the fracture site to avoid sparing in such conditions [10]. On the other hand, CPB may not work in conditions where the fibrotic changes associated with the wound healing process compromise the potentiality of the space under clavipectoral fascia. Such conditions include delayed presentation of fracture, nonunion, malunion, revision surgeries, or implant removal surgeries.

We believe that the deposition of LA over the periosteal collar of the subcutaneous bone, such as the clavicle, does not require ultrasound guidance. Instead, it is sufficient to palpate either end with fingers and deposit LA over the periosteum of the clavicle to achieve the desired results of the ultrasound-guided CPB. In our institution, we have been using the landmark-guided CPB with the same desired efficacy with three injections: first on the medial end, second on the lateral end, and third over the fracture site of the clavicle (Figure 2, panel B). In our patient, we used both ultrasound-guided and landmark-guided CPB separately with equianalgesic efficacy for awake bilateral clavicle surgery. The efficacy of the landmark technique can prove to be an economic benefit, especially in resource-poor settings.

LA injected into the subcutaneous plane during the CPB can cause a cutaneous blockade above the clavicle through the diffusion of LA [14]; otherwise, additional supplementation with SCNB [45] or subcutaneous LA infiltration over the incision site will be sufficient. However, further studies are needed to clarify the cutaneous distribution of sensory blockade provided by the CPB. The adrenaline-containing LA solution used in our patient helped prolong the analgesic duration, achieve a clean and bloodless surgical field, and reduce total blood loss. We confirmed the required dermatomal coverage using pinpricks before shifting our patient inside the operating room, where the operating surgeon reconfirmed it using blunt forceps prior to the incision. Our patient remained comfortable throughout the surgery, except for the slight pain experienced during an unexpected lateral extension of incision on the right side that required an additional infiltration by the surgeon and an intravenous bolus (60 micrograms) of fentanyl. Such a change in the surgical plan at the eleventh hour poses a challenge to the growing interest in site-specific and procedure-specific RA. Despite detailed discussion with the surgical team, one must be aware of such possibilities.

CPB creates a field block around the clavicle without causing the sensorimotor alterations of the whole upper extremity. It enables the patient to use both upper limbs for independent routine activities such as eating or drinking. Our patient not only moved both upper extremities immediately after the surgery but also held a glass of juice with both hands in the recovery room postoperatively. He was discharged from the recovery room in less than an hour with a reported 10/10 satisfaction score with the block.

## Conclusions

Bilateral CPB is a viable option for providing surgical anesthesia for bilateral CF requiring early surgical intervention. It can be performed using landmark guidance with an equianalgesic profile as ultrasound guidance in an emergency or resource-poor setting. CPB can be a phrenic-sparing, motor-sparing, opioid-sparing, and procedure-specific RA technique for CF surgeries. In addition, it can be considered an effective alternative RA technique for clavicle surgery in suspected or confirmed brachial plexus injury. CPB is devoid of the risk of pneumothorax and polypharmacy-related postoperative complications such as nausea, vomiting, cognitive dysfunction. It is suitable for the Enhanced Recovery After Surgery (ERAS) protocol, as it favors early mobility and discharge. However, prospective studies with larger samples are needed to elucidate the distribution of sensory blockade, analgesic efficacy, and clinical safety of CPB.
